# Supplementary material for: Modelling associated factors of maternal age at first birth in Ethiopia: Gamma regression approach
Source: PLoS One. 2026 Apr 13;21(4):e0346504. doi: 10.1371/journal.pone.0346504 (PMC13075722; doi:10.1371/journal.pone.0346504)
Supplement: Attachment — Submitted filename: PLOS One- Maternal Age Review.pdf [file pone.0346504.s002.pdf]

Review Comments to the Author

Thank you for the opportunity to review this manuscript titled "Modelling Determinants of Maternal Age at First Birth in Ethiopia: Gamma Regression Approach." The study investigates an important demographic and public health issue by analyzing determinants of maternal age at first birth using data from the 2019 Ethiopia Mini DHS. While the topic is highly relevant, and the modeling approach is technically appropriate, the manuscript requires significant improvements in clarity, interpretation, and methodological justification before it is ready for publication.

1. Scientific and Technical Soundness

The analysis is largely methodologically sound. The Gamma regression model is an appropriate choice for modeling positively skewed continuous data such as maternal age at first birth. The use of both Generalized Linear Models (GLM) and Generalized Linear Mixed Models (GLMM) is commendable, as is the inclusion of both maximum likelihood and Bayesian estimation techniques with appropriate diagnostic checks ($\hat{R} < 1.1$, high ESS, low MCSE).

However, the justification for excluding key socio-demographic variables (e.g., education, marital status, occupation) due to timing of measurement is a concern. These are important predictors of maternal age at first birth and their exclusion may introduce bias. At the very least, a deeper discussion of potential omitted variable bias and its implications on results should be provided.

Additionally, a brief rationale for choosing Gamma regression over time-to-event models (e.g., Cox proportional hazards, AFT models) would strengthen the manuscript, particularly given the nature of the outcome (age at first birth).

2. Data and Availability

The manuscript complies with PLOS ONE's data availability policy. The data are drawn from a publicly accessible, de-identified source—the 2019 Ethiopia Mini DHS via the Measure DHS program. The authors clearly state that all datasets are fully available without restriction, which meets the journal's requirements. However, they should provide the exact link to the data access page and any relevant dataset identifiers to facilitate reproducibility.

3. Statistical Rigor and Interpretation

The statistical analysis is rigorous, with well-justified use of a Gamma distribution and appropriate model diagnostics. The dual estimation approach (MLE and Bayesian) is a notable strength and enhances confidence in the robustness of the findings. Diagnostic techniques such as posterior predictive checks, convergence monitoring, and model fit metrics are all appropriately used.

That said, the interpretation of model results is relatively limited. The discussion would benefit from more explanation of why particular regional or religious groups are associated with earlier or later age at first birth. Linking statistical results to sociocultural or health system factors in Ethiopia would give the analysis greater depth and relevance.

Also, while model fit statistics like AIC and BIC are mentioned, it would be helpful to clearly present them in a summary table and offer a more direct comparison of the two models (GLM vs. GLMM) to guide reader understanding.

4. Writing Quality and Clarity

This is a major weakness of the manuscript. The English language quality is not sufficient for publication in its current form. The paper contains frequent grammatical errors, awkward phrasing, incorrect syntax, and unclear sentence structures that significantly hinder readability. For example:

- “The teenage a woman is…” → should be “The younger a woman is…”
- “mor far” → should be “more distant”
- Phrases such as “This issue is linked to various negative impacts…” are vague/unclear and redundant.

The Introduction and Background sections are too long and repetitive. Several paragraphs reiterate the same facts about early childbirth and maternal health risks, often using slightly different wording. This redundancy should be minimized to improve focus and clarity.

The Bayesian estimation section is also overly technical for a general readership. Simplifying the language and breaking up long blocks of equations and dense paragraphs will improve accessibility.

A professional English-language editor or native speaker with expertise in scientific writing should be engaged to revise the manuscript thoroughly.

5. Figures, Tables, and Visual Aids

Some of the figures (e.g., Figure 1a–1c, Figure 2) are not clearly labeled or well integrated into the discussion. Each figure should be accompanied by a complete, self-contained caption and referenced explicitly in the text with interpretation. The density plots and boxplots are helpful, but their readability can be improved.

Additionally, a summary table of model fit statistics (AIC, BIC, LOO, etc.) and key coefficient estimates with credible/confidence intervals would strengthen the presentation.

6. Ethics and Compliance

The authors state that the study received appropriate ethical approval from the IRB of ICF, with data anonymized to prevent identification. This aligns with ethical standards for secondary data analysis.

No concerns were identified regarding dual publication, misconduct, or research ethics.

Conclusion and Recommendation

This study has the potential to make a valuable contribution to the literature on early childbirth and maternal health in Ethiopia. The modeling approach is sound, and the use of both classical and Bayesian estimation adds methodological richness.

However, major revisions are required before the manuscript is suitable for publication. These include:

- Substantial improvement of English language and clarity
- More comprehensive discussion of excluded variables and potential biases
- Stronger interpretation of statistical results in their social and policy context
- Clearer presentation of model comparison and fit statistics

I encourage the authors to revise and resubmit the manuscript with these improvements. Once addressed, this work could make a meaningful contribution to public health research and policy in Ethiopia and similar settings.
