# Supplementary material for: Modelling associated factors of maternal age at first birth in Ethiopia: Gamma regression approach
Source: PLoS One. 2026 Apr 13;21(4):e0346504. doi: 10.1371/journal.pone.0346504 (PMC13075722; doi:10.1371/journal.pone.0346504)
Supplement: Attachment — Submitted filename: Response to Reviewers.docx [file pone.0346504.s003.docx]

**Response to Reviewers**

| **Reviewer Comment** | **Author Response** |
| --- | --- |
| **Reviewer #1 – Major Concerns** |  |
| Variable selection rationale: Exclusion of education, marital status, and occupation may introduce omitted variable bias. | We thank the reviewer for highlighting this point. Our study focused on maternal characteristics directly influencing first birth timing. To address this concern, we conducted a sensitivity analysis including education, marital status, and occupation (results section, Table 5). Results show that their inclusion small changes in magnitude and does not change direction in the associations between region, religion, and residence with the response variable maternal age at first birth. We also clarified the limitations of variable exclusion in the discussion (page 46 under strengths and limitations sub title). |
| Statistical modeling decisions: Insufficient justification for Gamma regression; lack of formal goodness-of-fit tests and rationale for log link. | We now provide formal goodness-of-fit assessments comparing Gamma, Normal, log-normal, and Weibull distributions, including AIC values and Q-Q plots (Table 2, Figure 5). Results indicate that the Gamma distribution with log link best fits the strictly positive, right-skewed outcome. The log link ensures positive predicted values and allows multiplicative interpretation of covariate effects, consistent with standard practice. |
| Focus on women who had given birth only: Excluding women without births may introduce selection bias; consider survival analysis or descriptive comparison. | We acknowledge this limitation and the study objective is to see only mothers who had already give birth. While survival analysis could accommodate censoring, the objective of this study is to see only women who had given birth and associations with regional religious and residential factors; thus, our approach remains appropriate for cross-sectional analysis. Results are interpreted as associations among women who have already had first birth. |
| Causal inference limitations: Avoid causal language; emphasize associations. | All statements throughout the manuscript have been revised to use associational language (e.g., associated with instead of “determines”) and to acknowledge potential unobserved confounders. |
| Theoretical framework: Lack of clear mechanisms linking region, religion, and residence to maternal age at first birth. | We strengthened the theoretical framework in the introduction (pages 4–5), discussing potential mechanisms. |
| **Reviewer #1 – Minor Issues** |  |
| Presentation of results: Tables 3 and 4 are dense. | Tables have been split: key coefficients and 95% CIs appear in main manuscript (Table 7, 8), detailed diagnostics (R̂, ESS, MCSE) also included avoiding more tables. Coefficient plots especially trace plot for Afar region and for intercept were added for improved readability (Figure 6). |
| Literature review organization: Currently chronological; suggest thematic. | The literature review is reorganized thematically into: (i) regional variations, (ii) methodological approaches, and (iii) determinants, highlighting research gaps (pages 3-4). |
| Statistical terminology inconsistencies: e.g., “gamma regression,” “gamma family GLM,” “dispersion parameter,” “mixed-effects.” | Terminology standardized: **“**Gamma regression with log link”, “dispersion parameter”, and “random effects**”** are used consistently throughout the manuscript. |
| Grammatical errors and typographical issues. | Manuscript has been carefully proofread. Redundant sentences removed; capitalization of “Mini EDHS 2019” standardized; complex methodology sentences simplified. |
| Figures quality and presentation: Low resolution, poor labels. | Figures have been recreated at high resolution, with clear axis labels, consistent font size (10–12 pt), legends placed outside plot areas, and enhanced readability (Figures 1-7). |
| Specific recommendations: Introduction, methods, results, discussion phrasing. | All recommended phrasing improvements have been incorporated. Results now report adjusted percent differences with 95% CIs. Discussion contextualizes findings in social and policy terms. |
| **Reviewer #2 – Major Points** |  |
| Exclusion of socio-demographic variables may introduce bias. | See response to Reviewer #1, Major Concern 1. Sensitivity analysis confirms main associations are robust; limitations are explicitly discussed. |
| Rationale for Gamma regression vs. survival models. | Outcome is observed age at first birth among women who already had a birth. Gamma regression appropriately models a strictly positive, right-skewed variable. Survival models require longitudinal follow-up and censored data. So the objective of the study is to determine magnitude and regional religious and residential associations with women’s first age at birth who had already had birth. |
| Data availability: Provide exact link and dataset identifiers. | Revised Data Availability Statement now includes direct DHS link and dataset identifiers (page 8) for reproducibility. |
| Interpretation of statistical results: Need more context. | Discussion now elaborates on why specific regions and religious groups are associated with earlier or later first birth, drawing on cultural, educational, and health system factors (pages 32-40). |
| Presentation of model fit statistics: GLM vs GLMM. | Added a summary table of AIC, BIC, LOO, WAIC comparing GLM, GLMM and distributional gamma regression (Table 3), with clear interpretation. |
| Writing quality and clarity: English issues, redundancy, overly technical Bayesian section. | Manuscript thoroughly revised for clarity and readability; Bayesian estimation section simplified with clear explanations; professional scientific editor engaged. |
| Figures, tables, and visual aids. | Figures updated for resolution and clarity; table summarizing key coefficients and CIs added; supplementary tables include detailed diagnostics. |
| Ethics and compliance. | Ethical statement now justified. |
| **Reviewer #3** |  |
| Need stronger justification for model selection, clearer GLM vs. GLMM comparison, expanded diagnostics, and more actionable public health discussion. | We thank the reviewer. (i) Justification for Gamma regression strengthened with formal goodness-of-fit comparisons and Q-Q plots (Figure 5, Table 3); (ii) clear GLM vs. GLMM comparison added (Table 3), highlighting fit and interpretation of random effects; (iii) expanded diagnostics provided, including posterior predictive checks, trace plots, and convergence statistics (Table 7-8); (iv) discussion now emphasizes policy-relevant implications, offering actionable recommendations for regional, religious, and rural urban disparities in maternal age at first birth. |
| **Reviewer #4** |  |
| Manuscript resembles a thesis; methodology should be concise; figures require high resolution, labeling, and informative captions; demonstrate model assumptions; consider resampling for DHS sampling bias; improve grammar and clarity. | We thank the reviewer. (i) Methodology section streamlined to focus on study-specific application of Gamma regression, with references to standard texts; (ii) all figures recreated at high resolution with clear axis labels, legends outside plots, and informative captions (Figures 1-7); (iii) assumption checks added, including Q-Q plots, residual analyses, and discussion confirming Gamma regression assumptions are met; (iv) resampling via bootstrap conducted to assess potential DHS sampling imbalance, confirming robustness; (v) manuscript underwent thorough language editing to improve grammar, clarity, and readability. |
