# Supplementary material for: Modelling associated factors of maternal age at first birth in Ethiopia: Gamma regression approach
Source: PLoS One. 2026 Apr 13;21(4):e0346504. doi: 10.1371/journal.pone.0346504 (PMC13075722; doi:10.1371/journal.pone.0346504)
Supplement: Attachment — Submitted filename: Response_to_Reviewers_auresp_2.docx [file pone.0346504.s004.docx]

Response to Reviewers

| **Reviewers Questions** | Author Responses |
| --- | --- |
| Add quantitative interpretation of key effects in natural units (e.g., “≈ 0.9 years earlier” rather than “5 % lower”). | Quantitative interpretation is added accordingly |
| Report random-effect variance (σ² or ICC) explicitly to reinforce GLMM justification. | Reported accordingly |
| Check reference formatting some citations still lack year-parenthesis uniformity | Checked the reference accordingly |
| Figures 2 & 3: The labeling and captioning are inconsistent with the figure content. Figure 2 needs category labels, axis units, and a legend; Figure 3’s caption should be corrected to reflect that only regional frequencies are shown, not residence or religion. The color scheme should be simplified and consistent across figures. ALso check other figures too for inconsistencies. | Corrected accordingly |
| Reviewer #4: The manuscript has improved considerably; however, please review the punctuation and formatting of the equations for consistency. Some equations need proper alignment and spacing, and equations that conclude a sentence should end with a full stop, while those followed by a continuation should use a comma. Ensuring this consistency will enhance the overall readability and professionalism of the manuscript. | Corrected accordingly |
| **Editor Comments: Please remove the headings and subheadings from the objectives section and integrate those listed items into the introduction as a coherent sentence.**  **There are several irrelevant subheadings in your methods section that need to be formatted properly. You need to combine Sections 2 and 3 into a single section titled 'Data and Methods'. Then, Section 3 should become Results, and Section 4 should be Discussion to ensure a logical flow of ideas. Substantive language editing is needed. All these issues to be addressed clearly.** | Corrected accordingly |
