# Supplementary material for: Modelling associated factors of maternal age at first birth in Ethiopia: Gamma regression approach
Source: PLoS One. 2026 Apr 13;21(4):e0346504. doi: 10.1371/journal.pone.0346504 (PMC13075722; doi:10.1371/journal.pone.0346504)
Supplement: Attachment — Submitted filename: Response_to_Reviewers_auresp_3.docx [file pone.0346504.s005.docx]

Manuscript Title: *Modelling Determinants of Maternal Age at First Birth in Ethiopia: Gamma Regression Approach*

Journal: PLOS ONE

Corresponding Author: **Adimias W. Agegnehu**

We sincerely thank the Academic Editor and the reviewers for their constructive comments. Their suggestions significantly improved the clarity, methodological transparency, and interpretation of the manuscript. All comments have been carefully considered and addressed in the revised manuscript. Detailed responses are provided below.

**A. Responses to Academic Editor Comments**

| Editor Comment | Author Response | Location in Revised Manuscript |
| --- | --- | --- |
| Definition of Target Population and Exclusion of Censored Observations | We thank the editor for highlighting this important methodological issue. The analysis indeed focuses only on women who had experienced a first birth by the time of the survey. To avoid misinterpretation, we revised the manuscript to clarify that all estimates apply **only to women who had already experienced first birth at the time of the survey** rather than to all women in Ethiopia.  Statements implying unconditional population-level inference were removed or rephrased throughout the Abstract, Results, and Conclusion sections. Additionally, a **new limitation subsection** has been added explaining that women without a first birth represent **right-censored observations in a time-to-event framework** and that excluding them may bias mean estimates toward younger ages. We also added a discussion comparing our estimates with the **DHS median age at first birth obtained using survival analysis** and clarified why a regression approach was chosen for conditional modeling of observed ages. | Abstract, Results, Conclusion revised; new subsection **“Study Limitations”** added in Discussion |
| Survey Design and Sampling Weights | We appreciate the editor’s suggestion. The Methods section has been revised to clearly describe the **two-stage stratified cluster sampling design used in the 2019 Ethiopia Mini Demographic and Health Survey**. Sampling weights provided by the DHS were incorporated in the analysis to ensure representativeness. We also clarified how clustering and stratification were handled in variance estimation. Additional methodological details were added to allow reproducibility of the analysis. | Methods section – “Data Source and Survey Design” |
| Interpretation and Scope of Inference | We agree that causal interpretations should be avoided. The manuscript has been revised to replace causal terminology such as “determinants” with **“factors associated with maternal age at first birth.”** Policy interpretations have been moderated to reflect the **cross-sectional nature of the data and the conditional sample of women who had experienced first birth**. Statements regarding religion and regional differences were rewritten to emphasize **associations rather than causal relationships**. | Title, Abstract, Discussion revised |
| Omitted Variable Bias and Model Specification | We appreciate this important comment. The Discussion section has been expanded to explicitly acknowledge the potential for **omitted variable bias**, particularly due to the exclusion of variables such as education, marital status, and occupation. These variables may be endogenous or measured after the timing of first birth. The revised manuscript now clarifies that estimated associations for region and religion may partly reflect **unmeasured confounding factors**. This limitation is discussed in greater detail. | Discussion – “Limitations and Potential Confounding” |
| Model Comparison and Diagnostics | Following the editor’s recommendation, we added a **comprehensive model comparison table** summarizing model performance across estimation frameworks. The table reports **AIC, BIC, and log-likelihood for classical models** and **LOO and expected log predictive density (ELPD) for Bayesian models**. Convergence diagnostics including **R-hat and effective sample size** are also reported. We further included **posterior predictive checks** to evaluate model adequacy. The preferred model is now clearly identified and justified based on predictive performance and goodness-of-fit. | Results section – new **Model Comparison Table** and **Diagnostics subsection** |
| Balance Between Mathematical Derivation and Substantive Interpretation | We agree that the detailed derivations may be excessive for an applied demographic analysis. Accordingly, the extensive mathematical derivations of likelihood functions, score equations, and Hessian matrices have been **moved to the Supplementary Material**. The main text now focuses on a concise description of the models, estimation procedures, and interpretation of parameters. This change substantially improves readability and aligns the manuscript with conventions of applied statistical research. | Methods section streamlined; derivations moved to **Supplementary Appendix** |
| Data Quality Clarification (minimum age at first birth) | We verified the DHS dataset and confirmed that the minimum reported age at first birth is **10 years**, which reflects rare but documented early childbirth cases rather than data entry errors. A clarification has been added to the Results section explaining that such values represent extremely early births and should be interpreted cautiously. | Results section – Data description |
| Data Availability Statement | The Data Availability Statement has been revised to comply with the journal’s policy. The dataset used in this study is publicly available through the **Demographic and Health Surveys (DHS) Program** upon registration and request. The revised manuscript now includes the appropriate statement and access link. | Data Availability Statement revised |

**B. Responses to Reviewer Comments**

| **Reviewer 3:** Reviewer Comment | Author response |
| --- | --- |
| Figure 3 appears to show only regional distribution of participants and duplicates information in Table 1. The figure does not illustrate maternal age at first birth patterns. | We thank the reviewer for this observation. We agree that the original figure did not provide additional analytical insight. Therefore, **Figure 3 has been removed** to avoid redundancy, as the information is already presented in Table 1. |
| **Reviewer 4:** Reviewer Comment |  |
| The manuscript is applied rather than methodological. Mathematical derivations of estimation procedures should be reduced. | We appreciate the reviewer’s suggestion. The manuscript has been revised to **substantially reduce the mathematical derivations in the main text**. The modeling framework is now presented in a concise form focusing on model specification and interpretation. Detailed derivations related to maximum likelihood estimation and Bayesian inference have been moved to **Supplementary Material**. This revision improves readability and better aligns the paper with applied statistical literature. |
